# Hemiscrotal agenesis: a novel phenotype of a rare malformation

**DOI:** 10.1186/s12887-020-02424-y

**Published:** 2020-11-28

**Authors:** Mohamed Mansy, Mostafa Kotb, Yasmine Abdelmeguid, Shaymaa Raafat, Marwa Abdelaziz

**Affiliations:** 1 Pediatric Surgery, Port Said Faculty of Medicine, Port Said, Egypt; 2 Department of Pediatric Surgery, Alexandria Faculty of Medicine, 21615 Alexandria, Egypt; 3 Pediatric Endocrinology, Alexandria Faculty of Medicine, Alexandria, Egypt; 4 Pathology, Alexandria Faculty of Medicine, Alexandria, Egypt

**Keywords:** Case report; Hemiscrotal agenesis; Monodactylous limb

## Abstract

**Background:**

Hemiscrotal agenesis (HSA) is an exceedingly rare congenital anomaly in scrotal development. It is characterized by unilateral absence of scrotal skin with intact midline raphe. In the English literature, only seven patients were diagnosed with HSA. Herein, we report a 14-month-old boy with HSA, unilateral cryptorchidism and a perineal skin tag. Additionally, the patient had a monodactylous limb, unilateral cerebellar hypoplasia, and a cardiac septal defect.

**Case presentation:**

A 14-month-old boy presented with right HSA and ectopic scrotal skin in the right perineal region. Extra-genital examination showed right monodactylous lower limb, without dysmorphic facial features or any other skeletal anomalies. His karyotype was 46, XY, while his hormonal profile showed prepubertal LH and FSH. Skeletal survey showed right monodactylous lower limb (with only a big toe which had 2 phalanges) and normal spine alignment. A previous echocardiography was done and showed a small muscular ventricular septal defect (VSD) that closed on follow-up. Magnetic resonance imaging of the brain showed posterior fossa malformation. The patient had his right testis fixed in the right scrotum. The pathological examination of the perineal lesion showed fibro-epithelial polyp (skin tag), with no testicular tissue or atypia.

**Conclusion:**

We believe that this is the first case to be reported with hemiscrotal agenesis and ipsilateral cryptorchidism, associated with a perineal skin tag, unilateral monodactylous lower limb on the same side, unilateral cerebellar hypoplasia, and VSD. Interestingly, further genetic analysis is required to reach a final diagnosis. However, regrettably, advanced molecular diagnostic studies for this patient is not available in our country.

## Background

Hemiscrotal agenesis (HSA) is a very rare congenital anomaly in scrotal development. It is characterized by unilateral absence of scrotal skin with intact midline raphe [1]. In the English literature, only seven patients were diagnosed with HSA [1–7]. Occasionally, this anomaly can be associated with extra-genital anomalies as a part of a syndrome, which supports the existence of a genetic background in these patients [2].

This anomaly is proposed to be due to primary failure of labioscrotal folds development or secondary to localized 5-alpha-reductase type 2 deficiency or androgen insensitivity [1, 2]. Herein, we report a 14-month-old boy with HSA, unilateral cryptorchidism and a perineal skin tag. Furthermore, the patient had a monodactylous limb, unilateral cerebellar hypoplasia, and a cardiac septal defect.

## Case presentation

**Fig. 1:**
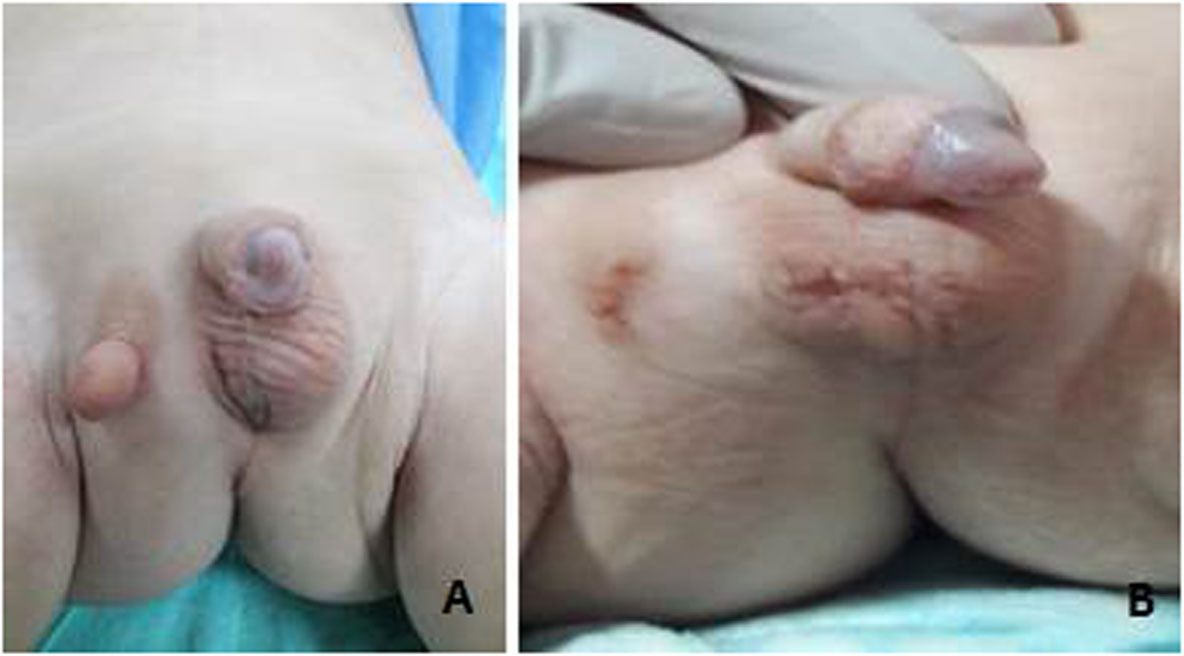
**a**. A preoperative photo showing absence of scrotal rugae on the right side, ipsilateral cryptorchidism and abnormal external tissue on the right side of the perineum. **b**. The same patient 6 months after the ochiopexy

**Fig. 2:**
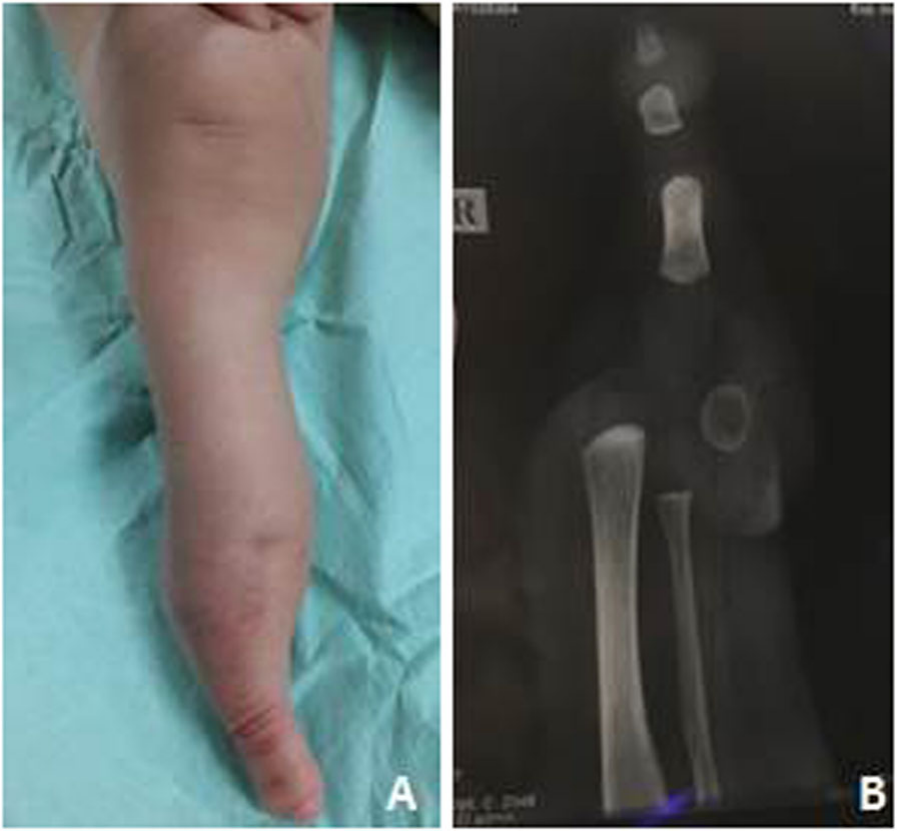
**a**. Limb examination showed right monodactylous lower limb. **b**. Skeletal survey showed right monodactylous lower limb with only a big toe with 2 phalanges

A 14-month-old boy was referred to our hospital for atypical genitalia. He is the only child, born to non-consanguineous parents by cesarean section after an uneventful pregnancy. Examination of external genitalia revealed absence of scrotal rugae on the right side, ipsilateral cryptorchidism and abnormal external tissue on the right side of the perineum (Fig. 1). The right testis was palpable in the inguinal region, the left testis was in the scrotum with intact midline raphe, and his penile length was 4.8 cm (normal for age). Ultrasonography showed that both testes were normal in size and echotexture. Extra-genital examination showed right monodactylous lower limb, normal tone and deep tendon reflexes. He had no dysmorphic facial features or any other skeletal anomalies. (Fig. 2a).

The karyotype of the boy was 46, XY, while his hormonal profile showed normal prepubertal LH and FSH (0.1, 0.4 mIU/ml respectively). After ß-hCG stimulation, serum total testosterone increased from 0.03 ng/ml to 3.51 ng/ml (normal basal serum total testosterone: up to 0.3, normal serum total testosterone after ß-hCG stimulation: more than 2-fold rise) and testosterone/dihydrotestosterone ratio was 8.54 ng/ml (normal: up to 30). Moreover, he had normal sertoli cell function which was assessed by measuring inhibin-B and anti-Müllerian hormone which were 158 pg/ml (normal: 91–163) and 23 ng/ml (normal: above 15), respectively.

In addition, work-up for associated extra-genital anomalies was done. Skeletal survey showed right monodactylous lower limb (with only a big toe which had 2 phalanges) (Fig. 2b) and normal spine alignment. This limb malformation resulted in motor developmental delay for which he started to stand supported with the help of orthopedic shoes, physical and occupational therapy. There was neither a family history of similar condition nor seizures, abnormal movements or ataxia. Associated intra-abdominal anomalies including renal system abnormalities were excluded by abdominal and pelvic ultrasonography. A previous echocardiography showed a small muscular ventricular septal defect (VSD) that closed on follow-up.

**Fig. 3:**
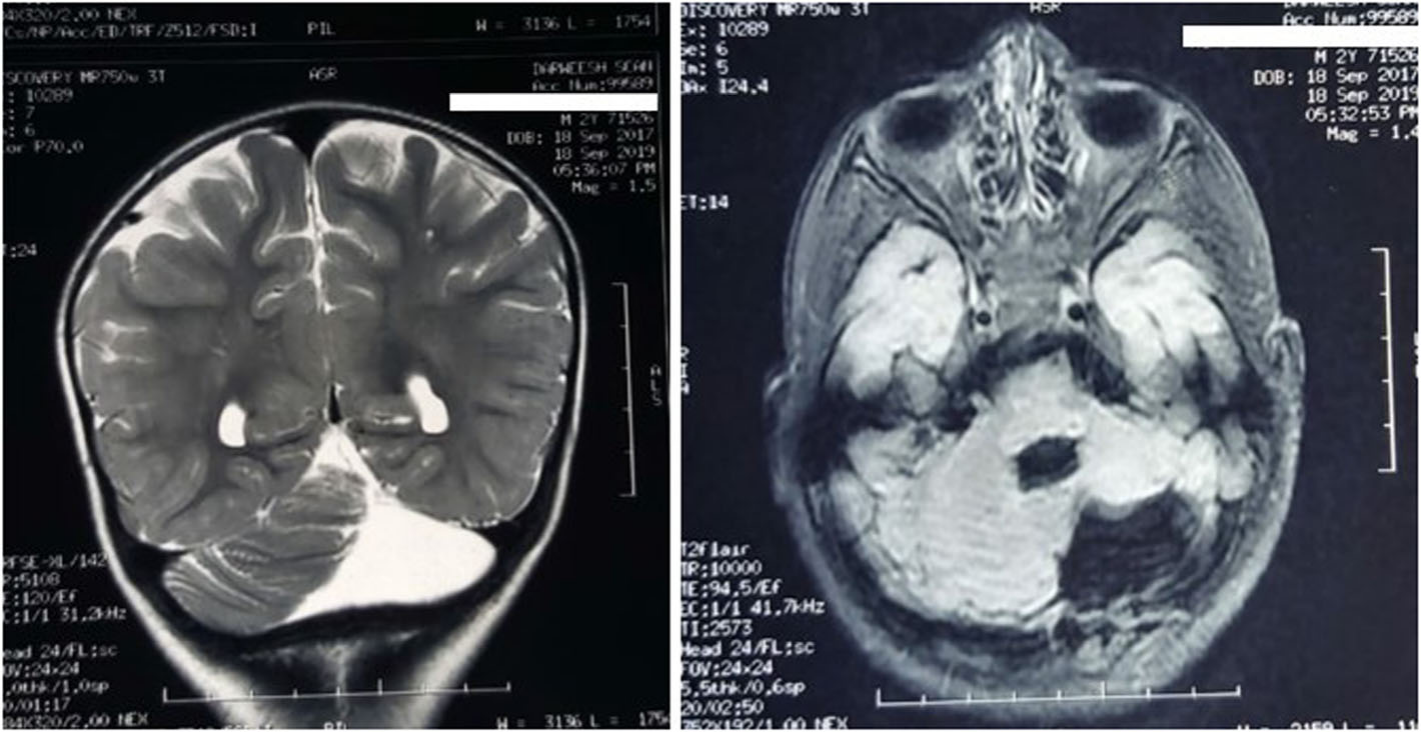
Magnetic resonance imaging of the brain revealed hypoplasia of the antero-superior compartment and total aplasia of the postero-inferior compartment of the left cerebellar hemisphere

Magnetic resonance imaging of the brain showed posterior fossa malformation in the form of dysplastic left cerebellar hemisphere with hypoplasia of its antero-superior compartment, total aplasia of its postero-inferior compartment and a small cleft traversing the inferior aspect of the right cerebellar hemisphere (Fig. 3). Despite that no seizures were reported, right focal epileptiform activity was detected by electroencephalogram. Audiological examination revealed bilateral normal function, and his ophthalmological examination was normal.

**Fig. 4:**
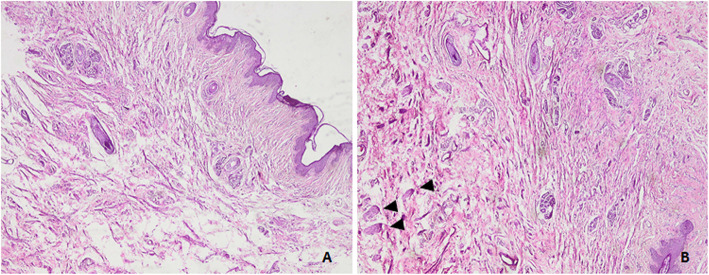
**a**. Scrotal skin showing corrugated skin surface composed of a thin layer of keratinized stratified squamous epithelium, overlying scattered slender smooth muscle bundles representing the superficial layer of dartos muscle (arrows). Scattered hair follicles and sweat glands are seen. No sebaceous glands are found. [H&E X40]. **b**. The deep reticular dermis shows few scattered thick smooth muscle bundles representing the deep layer of dartos muscle (arrowheads). Note the complete absence of subcutaneous fat. [H&E X40]

Intra-operatively, the patient had his right testis fixed in the right scrotum. Two biopsies were taken; one of them from the scrotal skin and the other from the perineal lesion. The pathological examination of the perineal lesion showed fibro-epithelial polyp (skin tag), with no testicular tissue or atypia (Fig. 4).

## Discussion

Scrotal agenesis either bilateral or unilateral is considered to be an extremely rare developmental anomaly of the scrotum [8]. During fetal life, scrotal development starts by formation of labioscrotal folds on both sides of the urogenital folds in the 7th embryonic week. Testosterone reaches its peak during the 10th gestational week and is converted by the enzyme 5-alpha reductase into more potent dihydrotestosterone (DHT), which induces virilization of the external genitalia [9]. At the 16th gestational week, fusion of the labioscrotal folds occurs to form the scrotum [1, 3].

Despite that the embryological development of the external genitalia is well known, the exact embryological etiology of scrotal agenesis is still unknown. Maldevelopment of labioscrotal folds is considered to be the cause of scrotal agenesis by either mechanical or endocrine disorders [8]. It is also proposed that localized partial androgen insensitivity and 5a-reductase type 2 deficiency may be the cause. However, enough DHT is produced to prevent labia formation without stimulation of scrotal development, as evidenced by the presence of the midline raphe [1–3, 9].

Although the number of HSA cases reported in literature is scarse, the clinical phenotype varies. Three of the reported cases were associated with ipsilateral cryptorchidism [2, 4, 5] and the remaining 3 had complete testicular descent [1, 3, 6]. Moreover, scrotal agenesis may present as an isolated anomaly or may be associated with a variety of other congenital defects such as face anomalies, growth retardation, cardiac anomalies, anterior ectopic anus, digit anomalies [1, 4, 10]. Of the reported cases with HSA, 1 patient had PHACE syndrome, 1 patient had Van der Woude syndrome, and 1 had cutis marmorata telangiectatica congenita, and hydronephrosis due to vesicoureteral reflux [3, 6, 7]. Only 1 patient had an island of scrotal tissue in the pubic tubercle region, in addition to HSA [4].

As regards limb anomalies associated with atypical genitalia, few patients were shown to have mutation in human gene HOXA13 and HOXD13. HOX genes encode a family of transcription factors that play a major role in the development of the central nervous system, axial skeleton, gastrointestinal and urogenital tracts, external genitalia, and limbs during embryonic development. Mutations in these genes cause a wide range of malformations. The most common amongst these malformations are hand-foot-genital syndrome caused by HOXA13 gene mutation, and synpolydactyly caused by HOXD13 mutation. The severity of the phenotypic manifestation of these diseases is variable, and depends on the exact mutation type [11–13]. Interestingly, the presence of HAS along with unilateral cerebellar hypoplasia and unilateral monadactylus limb possibly suggest a vascular disruptive origin and according to Stephens, a possible mechanical pressure effect on the developing fetus could be the implicating factor [14].

To our best knowledge, this is the first case to be reported with hemiscrotal agenesis and ipsilateral cryptorchidism associated with a perineal skin tag, unilateral monodactylous lower limb on the same side, unilateral cerebellar hypoplasia, and VSD. Interestingly, further genetic analysis is required to reach a final diagnosis. Unfortunately, advanced molecular diagnostic studies for this patient is not available in our country.

## Data Availability

Data sharing is not applicable to this article as no datasets were generated or analysed during the current study.

## Abbreviations

DHT: Dihydrotestosterone
HSA: Hemiscrotal agenesis
VSD: Ventricular septal defect
ß-hCG: ß-subunit of Human chorionic gonadotrophin
